# Case Report: Novel *IRF2BP2* variant in a Japanese patient with impaired B-cell differentiation, Th1 polarization, and systemic immune dysregulation

**DOI:** 10.3389/fimmu.2025.1662899

**Published:** 2025-10-30

**Authors:** Yushiro Endo, Tomohiro Koga, Shota Kurushima, Takuya Tomokawa, Hiroyuki Mishima, Koh-ichiro Yoshiura, Tadashi Matsumoto, Atsushi Kawakami

**Affiliations:** ^1^ Department of Immunology and Rheumatology, Division of Advanced Preventive Medical Sciences, Nagasaki University Graduate School of Medical Sciences, Nagasaki, Japan; ^2^ Department of Human Genetics, Atomic Bomb Disease Institute, Nagasaki University, Nagasaki, Japan; ^3^ Department of Human Genetics, Leading Medical Research Core Unit, Graduate School of Biomedical Sciences, Nagasaki University, Nagasaki, Japan; ^4^ Department of Human Genetics, Graduate School of Biomedical Sciences, Nagasaki University, Nagasaki, Japan

**Keywords:** IRF2BP2 variant, common variable immunodeficiency disorder (CVID), Th1 polarization, STAT1 signaling, B-cell differentiation

## Abstract

Interferon regulatory factor 2 binding protein 2 (IRF2BP2) is a transcriptional corepressor involved in immune regulation via IRF1-mediated interferon signaling inhibition. Pathogenic *IRF2BP2* variants are associated with common variable immunodeficiency, primarily affecting B-cell maturation. We report a 47-year-old female with immunodeficiency and systemic inflammation, including primary biliary cholangitis and unclassified arthritis, who was detected to carry a novel heterozygous *de novo* missense variant in the *IRF2BP2* gene (c.1663T>A; p. Cys555Ser). Immunophenotyping revealed naïve B-cell predominance, with a loss of memory B cells and impaired plasmablast differentiation, indicating late-stage disturbed B-cell differentiation/maturation. CD4^+^ T cells demonstrated Th1 polarization with reduced Th2 subsets, whereas Th17 and Treg populations exhibited no obvious changes. Considering that IRF2BP2 negatively regulates STAT1-driven transcription via IRF1 suppression, the observed Th1 polarization suggests improved STAT1 activity. This case underscores the combined humoral and cellular immune dysregulation due to IRF2BP2 dysfunction, expanding the clinical spectrum to encompass inflammatory phenotype.

## Introduction

Interferon regulatory factor 2 binding protein 2 (IRF2BP2) is a transcriptional regulator that plays a crucial role in immune response modulation (1). IRF2BP2 was originally identified as a corepressor of interferon regulatory factor 2 (IRF2) (2), which antagonizes the activity of IRF1, thereby attenuates interferon (IFN) signaling (3). Furthermore, IRF2BP2 has been demonstrated to interact with NFAT1 and other transcriptional regulators, participating in various cellular processes, including apoptosis, cell cycle, and cell differentiation (4–6). Recently, *IRF2BP2* variants have emerged as rare monogenic causes of common variable immunodeficiency disorder (CVID) (6–9). We here present a 47-year-old female from Japan with a novel heterozygous *IRF2BP2 de novo* missense variant, presenting with chronic sinusitis and airway infection with intermittent acute exacerbations as well as inflammatory phenotype, including primary biliary cholangitis (PBC) and unclassified arthritis.

## Case description

The patient experienced recurrent airway infection during early childhood. At 10 years old, laboratory evaluation revealed hypogammaglobulinemia characterized by markedly decreased immunoglobulin (Ig)M, IgG, and IgA levels, leading to a diagnosis of CVID. No family history of immunodeficiency or chronic inflammatory disease was reported; notably, both parents and siblings were healthy. Despite Ig replacement therapy (IgRT), the patient continued experiencing worsening episodes of chronic sinusitis and lower respiratory infections with bronchiectasis, as revealed on thoracic computed tomography. At 42 years old, the patient developed chronic hepatobiliary enzyme abnormalities. Laboratory test results demonstrated elevated levels of most hepatobiliary enzymes, with normal total bilirubin levels (**Supplementary Table S1**). Serum Ig levels remained significantly low for IgA and IgM, whereas IgG was slightly below the normal range under continuous IgRT. Autoantibody screening was negative for antinuclear and antimitochondrial M2 antibodies (**Supplementary Table S1**). Liver biopsy revealed chronic nonsuppurative destructive cholangitis characterized by dense lymphocytic infiltration around the interlobular bile ducts with epithelial invasion, accompanied by florid duct lesions and occasional epithelioid granulomas. Moreover, mild hepatocellular dropout and focal necrosis were noted. Azan staining demonstrated portal fibrotic expansion without bridging fibrosis, and orcein staining showed no copper-associated granule deposition. These findings, alongside the clinical course of cholestatic enzyme elevation and improvement following ursodeoxycholic acid therapy, were compatible with a PBC diagnosis, despite negative serological markers. At 44 years old, hospitalization was required owing to chronic productive cough exacerbation. Pulmonary function test demonstrated an obstructive impairment with an FEV1% of 63.9% and a preserved %VC of 84.6%. Bronchoalveolar lavage fluid (BALF) demonstrated a 49% lymphocyte fraction. Transbronchial lung biopsy revealed lymphocytic infiltration in the alveolar regions without granuloma formation or malignant findings. *Pseudomonas aeruginosa* was cultured from sputum and BALF samples. Considering these results and the presence of chronic productive cough, the findings were most consistent with a recurrent chronic airway infection rather than a granulomatous-lymphocytic interstitial lung disease, which is a severe noninfectious CVID complication (10). Antibiotic therapy improved the patient’s symptoms, further supporting the recurrent chronic airway infection diagnosis. Subsequently, the patient was referred to our department for evaluation of chronic arthralgia. Laboratory examination revealed an elevated C-reactive protein level (4.37 mg/dL); however, immunological and serological results, including infection-associated assays, were all negative (**Supplementary Table S1**). Although mild left radiocarpal joint swelling was noted, bilateral hand X-ray and Power Doppler ultrasonography revealed no bone destruction or synovitis, respectively. As her symptoms were mild, and she declined symptomatic treatment, the patient was followed up without any treatment for unclassified arthritis; X-ray revealed no evidence of progressive bone destruction.

**Figure 1:**
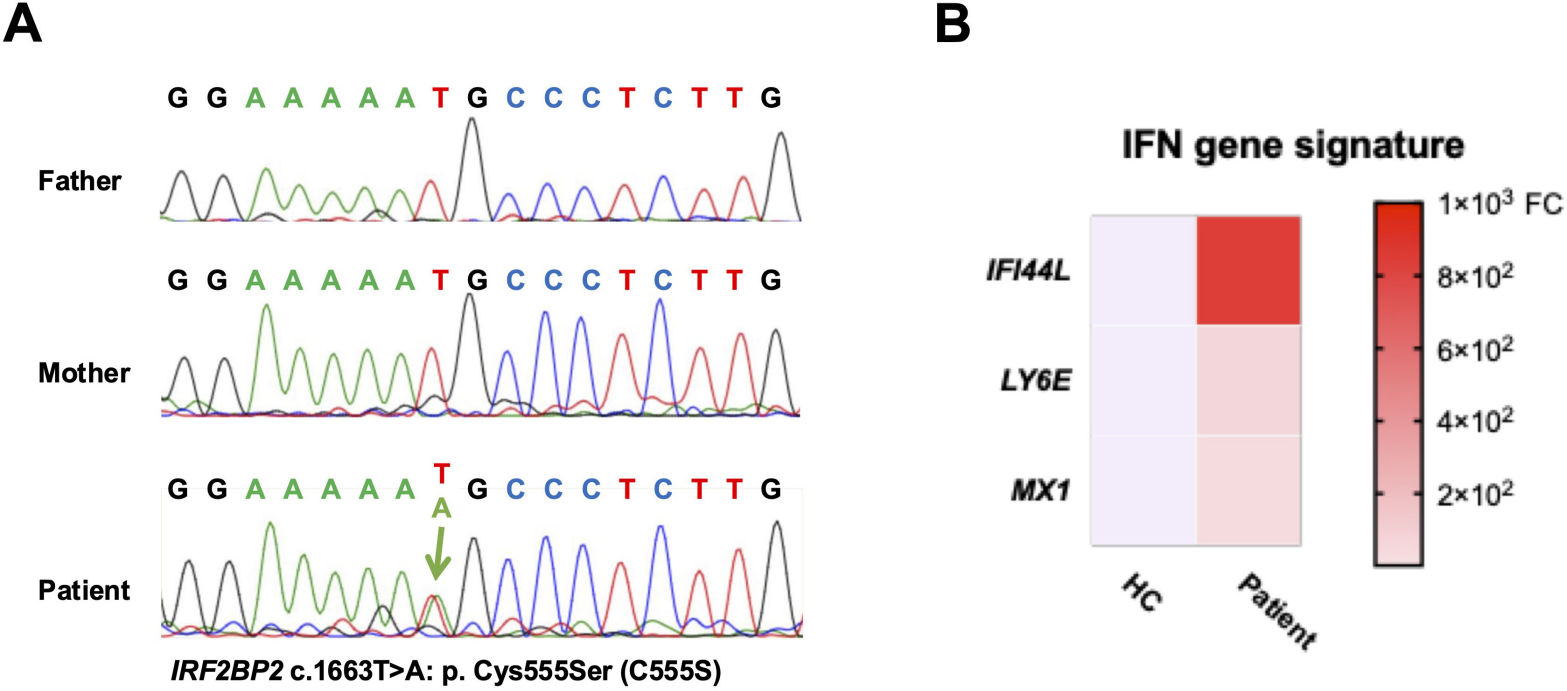
Identification of a novel *IRF2BP2* mutation. **(A)** Sanger sequencing chromatograms of the *IRF2BP2* gene showing a heterozygous *de novo* missense mutation (c.1663T>A; p. Cys555Ser) in the patient, which is absent in both parents. **(B)** Heatmap revealing the relative messenger RNA expression levels of *IFI44L*, *LY6E*, and *MX1* in PBMCs from the patient and a healthy control, as measured using quantitative polymerase chain reaction. Expression levels are normalized to *GAPDH* and calculated using the comparative CT method. Red indicates higher expression, and blue indicates lower expression relative to the control. FC, fold change.

As the patient presented with CVID and unexplained systemic inflammation, targeted next-generation sequencing of known CVID-related genes (*ICOS, TNFRSF13B, CD19, TNFRSF13C, MS4A1, CD81, CR2, LRBA, NFKB2, IL21, NFKB1, IKZF1, PLCG2, CTLA4*, and *MSN*) was performed at the Kazusa DNA Research Institute (Kisarazu, Chiba, Japan), which identified no pathogenic mutations. Subsequent whole-exome sequencing using trio samples (the patient and both her parents) was conducted as part of the Initiative on Rare and Undiagnosed Disease (IRUD) project in Japan (https://www.amed.go.jp/en/program/IRUD/), confirmed by Sanger sequencing. Genomic DNA was amplified using ExTaq DNA polymerase (Takara, Japan) with gene-specific primers: 5′-CTGCACCCTCTGCCACGA-3′ and 5′-TTAAGGGTAATCATTGGGT-3′ as the forward and reverse primers, respectively. Polymerase chain reaction (PCR) was performed under the following cycling conditions: 94°C for 10 s, 57°C for 20 s, and 71°C for 30 s, repeated for 35 cycles. PCR product was purified, and subsequently the same primers were employed for sequencing. The Sanger sequencing revealed a novel heterozygous *de novo* variant in the *IRF2BP2* gene at c.1663T>A: p. Cys555Ser (C555S) only in the patient (**Figure 1A**). Public databases, including ClinVar (https://www.ncbi.nlm.nih.gov/clinvar/) and gnomAD v4.1.0 (https://gnomad.broadinstitute.org/) have not reported this missense variant in the *IRF2BP2* gene. Regarding functional effect prediction, it was predicted to be “probably damaging” in PolyPhen 2 (http://genetics.bwh.harvard.edu/pph2/) with a score of 0.994 (sensitivity, 0.46; specificity, 0.96 in HumVar) and be “deleterious” in MutationTaster2025 (https://www.genecascade.org/MutationTaster2025/). No other genetic mutations explaining the immunodeficiency were observed.

Total messenger RNA from peripheral blood mononuclear cells (PBMCs) were isolated using the RNeasy Mini Kit (Qiagen) and subsequently synthesized cDNA using cDNA EcoDry Premix (Clontech, Mountain View, CA, USA) for PCR amplification. All primers and probes were obtained from Applied Biosystems (*IFI44L* [Hs00915292_m1], *LY6E* [Hs00158942_m1], *MX1* [Hs00895608_m1], and *GAPDH* [Hs02758991_g1]). The comparative CT method was employed for assessing gene expressions. The patient showed elevated type-1 IFN-linked (IFN-stimulated) gene expression, including *IFI44L*, *LY6E*, and *MX1*, compared with a healthy control (**Figure 1B**).

**Figure 2:**
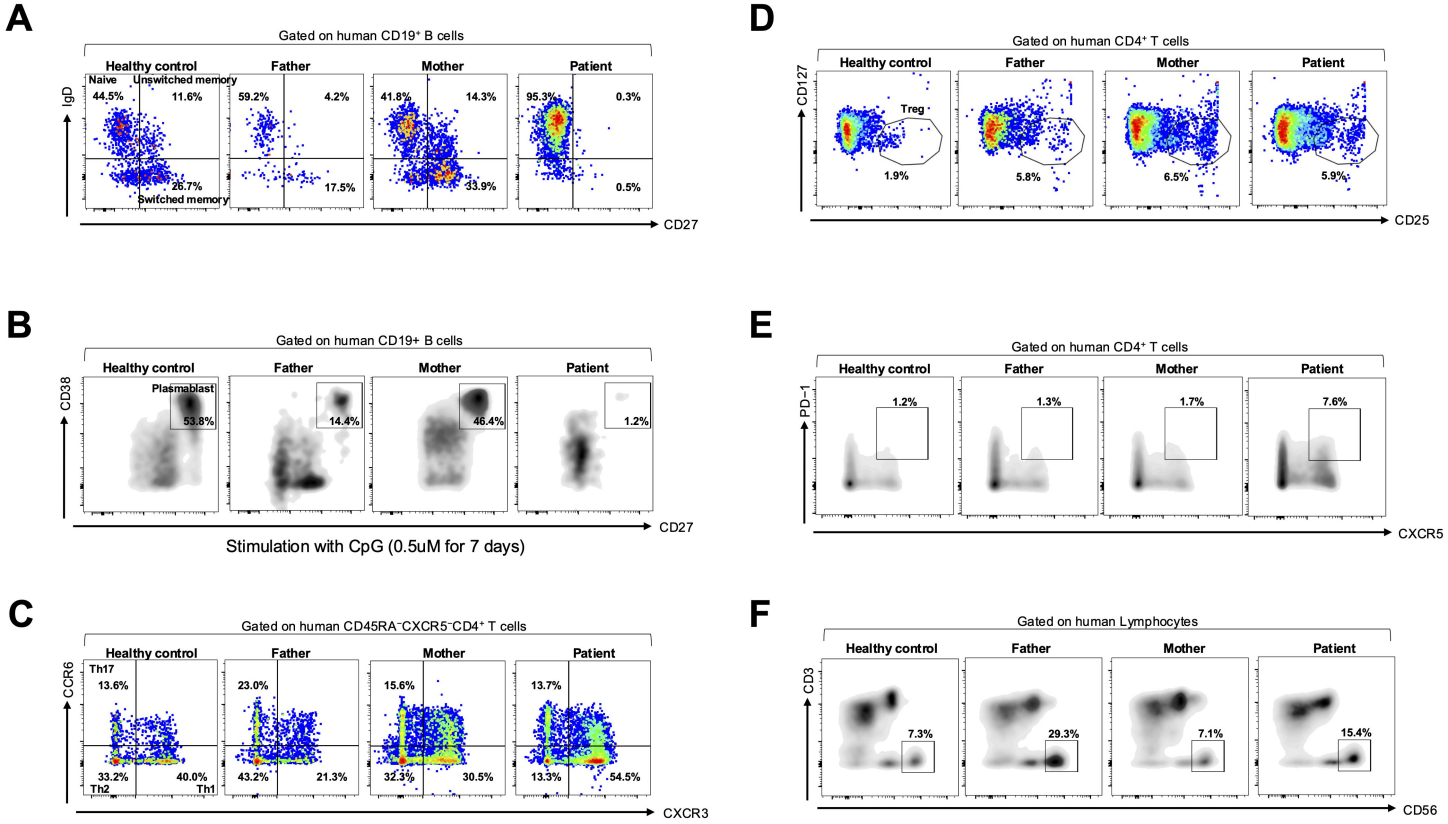
B-cell subset analysis and *in vitro* plasmablast differentiation assay. **(A)** Flow cytometric analysis of B-cell subsets gated on CD19^+^ B cells, including naïve (IgD^+^CD27^−^), switched memory (IgD^−^CD27^+^), and unswitched memory (IgD^+^CD27^+^) cells, in a healthy control, parents, and the patient. **(B)** *In vitro* plasmablast differentiation assay using PBMCs stimulated with CpG oligodeoxynucleotide (0.5 μM for 7 days), gated on plasmablasts (CD19^+^CD38^+^CD27^+^) among them. **(C)** Flow cytometric analysis of CD45RA^−^CXCR5^−^CD4^+^ helper T cells, including Th1 (CCR6^−^CXCR3^+^), Th2 (CCR6^−^CXCR3^−^), Th17 (CCR6^+^CXCR3^−^), **(D)** regulatory T (Treg; CD4^+^CD127^−^CD25^+^), **(E)** T follicular helper (Tfh; CD4^+^CXCR5^+^PD-1^+^), and **(F)** natural killer (NK; CD3^−^CD56^+^) cells in a healthy control, the parents, and the patient.

Subsequently, immune cell function was evaluated using flow cytometry. To isolate PBMCs, peripheral blood was processed with a lymphocyte separation medium (Corning). The isolated PBMCs were stained for 30 min at 4°C with fluorescent–conjugated antibodies. Flow cytometric acquisition was performed using an LSRII flow cytometer (BD Biosciences), and data were analyzed using FlowJo software version 7.6.1. The patient’s B cells were predominantly naïve (CD19^+^IgD^+^CD27^–^), with almost absent memory B cells, including unswitched memory (CD19^+^IgD^+^CD27^+^) and switched memory (CD19^+^IgD^–^CD27^+^), compared with the others (a healthy control and both her parents) (**Figure 2A**).

To assess differentiation into plasmablast, PBMCs were cultured as previously described (6, 11). Briefly, PBMCs were cultured in an RPMI 1640 medium with 10% fetal bovine serum and treated with a Toll-like receptor 9 agonist (unmethylated CpG oligodeoxynucleotide; Invivogen, San Diego, CA, USA) at a 0.50-μM final concentration. Cells were harvested following a 7-days incubation period and analyzed using flow cytometry. *In vitro* plasmablast differentiation revealed failure to differentiate into plasmablasts (CD19^+^CD38^+^CD27^+^) in the patient compared with the others (**Figure 2B**).

We focused on CD4^+^ T-cell subsets as laboratory examination revealed markedly decreased CD4^+^ lymphocyte levels (**Supplementary Table S1**). Regarding CD4^+^ T-cell subsets, the patient’s PBMCs showed a decreased percentage of T helper type 2 (Th2; CD45RA^−^CXCR5^−^CD4^+^CCR6^−^CXCR3^−^) and an increased percentage of T helper type 1 (Th1; CD45RA^−^CXCR5^−^CD4^+^CCR6^−^CXCR3^+^) cells compared with the others (**Figure 2C**). No remarkable difference in the percentage of T helper type 17 (Th17; CD45RA^−^CXCR5^−^CD4^+^CCR6^+^CXCR3^−^) and regulatory T (Treg; CD4^+^CD127^−^CD25^+^) cell populations was noted between them (**Figures 2C, D**). Furthermore, the patient’s PBMCs exhibited an increased percentage of T follicular helper (Tfh; CD4^+^CXCR5^+^PD-1^+^) cells compared with the others (**Figure 2E**). No remarkable difference in the percentage of natural killer (NK; CD3^−^CD56^+^) cells was observed between them (**Figure 2F**).

## Discussion

By underscoring concomitant B- and T-cell dysregulation caused by a novel heterozygous *de novo* variant in the *IRF2BP2* gene, this case expands the clinical and immunological spectrum of IRF2BP2-related immunodeficiency, suggesting that the novel C555S substitution influences severe phenotypes in immunodeficiency, including inflammatory features (PBC and unclassified arthritis), activated IFN–STAT1 pathway leading to Th1 polarization, and the Tfh expansion (**Supplementary Table S2**).

The p.C555S variant in exon 2 of *IRF2BP2* is linked to an amino acid change located in the RING domain of the protein, which is a critical region for its function as a transcriptional corepressor (9). A database search for the p.C555S variant yielded no results among 8, 380 healthy individuals in Japan (jMorp; https://jmorp.megabank.tohoku.ac.jp). Public prediction tools suggested that this variant is “probably damaging” (PolyPhen-2) and “deleterious” (MutationTaster), suggesting that replacing a conserved cysteine with serine can disturb the local conformation of the zinc-binding RING structure and compromise protein–protein interactions. Currently, the immunopathogenesis of IRF2BP2 dysfunction has mainly been attributed to B-cell defects. Familial cases of CVID with *IRF2BP2* variants demonstrate reduced memory B-cells levels and impaired B-cell differentiation into plasmablasts (6). Consistent with these findings, our patient exhibited a significant deficiency in memory B cells and a failure of CD19^+^ B cells to differentiate into plasmablasts *in vitro*, supporting the role of IRF2BP2 in late B-cell maturation.

Beyond disturbed B-cell differentiation and maturation, our patient also demonstrated Th1-type CD4^+^ T-cell profile predominance, which may be mechanistically associated with IFN–JAK–STAT1 signaling axis dysregulation due to IRF2BP2 dysfunction. IRF2BP2 was originally identified as a corepressor of IRF2 (2), which competes with IRF1 for binding to IFN-responsive gene promoters. While IRF1 promotes types I and II IFN-induced transcription, IRF2BP2, in complex with IRF2, normally represses this activation. Palmroth et al. reported that patients with *IRF2BP2* variants displayed systemic hyperactivation of STAT1 signaling, including constitutive and IFN-stimulated STAT1 phosphorylation in multiple leukocyte subsets, as well as increased expression of IFN-regulated genes (*IFIT1*, *IFIT3*, *IFI6*, *IDO1*, *CXCL9*, and *CXCL10*) (12). They further revealed that wild-type IRF2BP2 significantly suppressed STAT1-dependent transcriptional activity, whereas the mutant IRF2BP2 did not (12). Consistently, our patient demonstrated elevated expression of IFN signature genes (*IFI44L*, *LY6E*, and *MX1*), further supporting IFN–STAT1 pathway activation. Considering that STAT1 signaling is essential for CD4^+^ T-cell differentiation into Th1 cells (13), these findings suggest that the variant-induced loss of IRF2BP2 function causes enhanced STAT1 signaling, thereby promoting Th1 polarization. The increased proportion of Th1 cells observed in our patient aligns with this mechanism, further supporting the role of IRF2BP2 as a critical negative regulator of Th1 cell differentiation and subsequent IFN-mediated inflammation.

**Figure 3:**
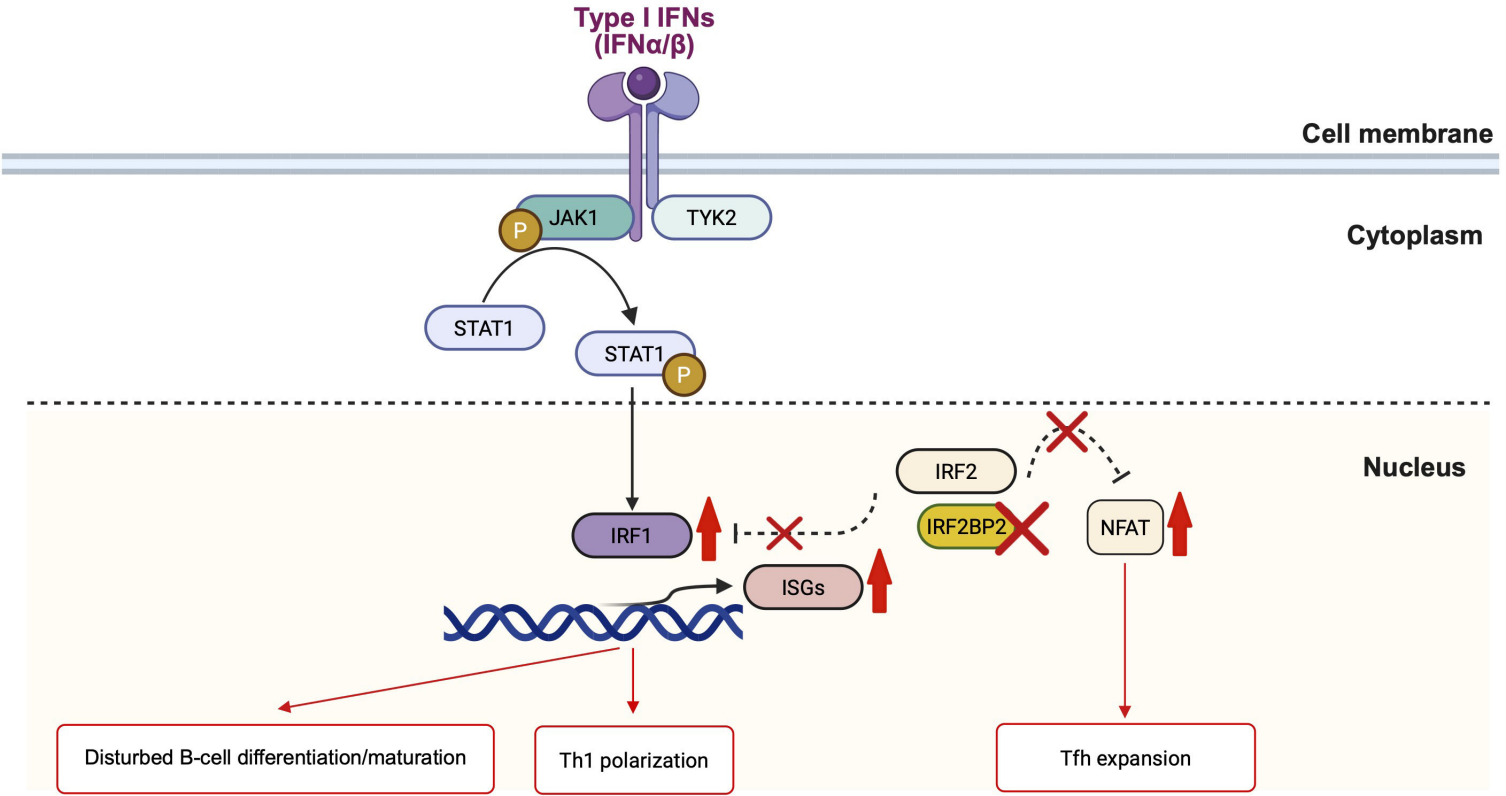
Schematic representation of IRF2BP2 signaling in immune regulation. The figure illustrating how IRF2BP2 serves as a nuclear transcriptional corepressor regulating IFN and NFAT-dependent gene expression. In the cytoplasm, type I IFNs (IFN-α/β) activate the JAK1–TYK2–STAT1 pathway, causing STAT1 phosphorylation and nuclear translocation. In the nucleus, phosphorylated STAT1 induces IRF1, which promotes IFN-stimulated gene (ISG) transcription. IRF2, alongside its corepressor IRF2BP2, generally antagonizes IRF1-mediated activation to maintain homeostasis. The loss of IRF2BP2 disrupts this inhibitory complex, causing excessive IRF1-driven ISG expression and enhanced STAT1 signaling, contributing to disturbed B-cell differentiation and Th1 polarization. In parallel, nuclear IRF2BP2 interacts with NFAT1 and suppresses its transcriptional activity; IRF2BP2 deficiency eliminates this restraint, causing NFAT-dependent Tfh expansion. IFN, interferon; ISG, interferon-stimulated gene; Tfh, T follicular helper cell. The figure illustration is created by BioRender (https://www.biorender.com/).

In addition to the Th1 polarization, our patient showed an increased frequency of circulating Tfh cells. IRF2BP2 has been demonstrated to directly bind to NFAT1, repressing its transcriptional activity (14). As NFAT signaling is crucial for Tfh differentiation and maintenance (15), the loss of IRF2BP2 function may lead to unregulated NFAT activation, thereby promoting aberrant Tfh expansion. Therefore, the elevated frequency of circulating Tfh cells observed in our patient offers further mechanistic evidence that IRF2BP2 dysfunction impairs late B-cell differentiation and contributes to immune dysregulation through Tfh expansion (**Figure 3**).

A recent report described that individuals with *IRF2BP2* variants develop immunodeficiency, frequently accompanied by inflammatory diseases (9), aligning with our patient who presented with CVID and systemic inflammation, including PBC and unclassified arthritis. Despite the absence of serological markers for rheumatoid arthritis, some patients with IRF2BP2 deficiency exhibited arthritis progression to destructive disease (16). However, our patient demonstrated mild arthritis that was conservatively managed without pharmacological intervention. Notably, our patient was diagnosed with PBC despite negative autoantibodies. The diagnosis was histologically confirmed by liver biopsy, which revealed portal inflammation consistent with PBC. This finding underscores that PBC may occur as a clinical phenotype of IRF2BP2 deficiency even in the absence of serological markers, underlining the significance of considering biopsy-proven PBC in patients with immunodeficiency exhibiting hepatobiliary abnormalities. As activated STAT1 signaling pathway and Tfh expansion are associated with autoinflammatory and autoimmune manifestations (17–20), patients with IRF2BP2 dysfunction may develop the inflammatory phenotypes.

## Conclusions

This case underscores the phenotype of IRF2BP2-related immunodeficiency with concomitant B- and T-cell dysregulation caused by a novel *IRF2BP2* variant. Notably, this case expands the clinical spectrum by identifying PBC as a novel IRF2BP2 deficiency phenotype. Clinicians should be aware of this association and consider PBC even in the absence of autoantibodies, particularly in patients with immunodeficiency.

## Data Availability

Datasets are available on request: The raw data supporting the conclusions of this article will be made available by the authors, without undue reservation.
