# Supplementary material for: Case Report: Novel IRF2BP2 variant in a Japanese patient with impaired B-cell differentiation, Th1 polarization, and systemic immune dysregulation
Source: Front Immunol. 2025 Oct 30;16:1662899. doi: 10.3389/fimmu.2025.1662899 (PMC12611837; doi:10.3389/fimmu.2025.1662899)
Supplement: Supplementary file 1 [file Table1.docx]

**Supplemental Table. Laboratory investigations in the present case**

|  |  | **Unit** | **Reference range** |  |  | **Unit** | **Reference range** |
| --- | --- | --- | --- | --- | --- | --- | --- |
| **T-Bil** | 0.6 | mg/dL | 0.4–1.5 | **TP** | 6.2 | g/dL | 6.6–8.1 |
| **AST** | 28 | U/L | 13–30 | **ALB** | 3.5 | g/dL |  |
| **ALT** | 18 | U/L | 7–23 | **IgG** | 419 | mg/dL | 861–1747 |
| **ALP** | 902 | U/L | 106–322 | **IgA** | <4 | mg/dL | 93–393 |
| **γ-GTP** | 123 | U/L | 9–32 | **IgM** | 19.2 | mg/dL | 50–269 |
| **LDH** | 187 | U/L | 124–222 | **ANA** | < 80 | times | < 80 |
| **BUN** | 9 | mg/dL | 8–20 | **AMA-M2** | Negative | NA | Negative |
| **Cre** | 0.45 | mg/dL | 0.46–0.79 | **Anit-LKM-1 antibody** | Negative | NA | Negative |
|  |  |  |  |  |  |  |  |
| **WBC** | 6500 | /μL | 3300–8600 | **RF** | <3.6 | IU/mL | <=15 |
| **Neutrophils** | 68.0 | % | 38.0–74.0 | **ACPA** | <0.6 | U/mL | <4.5 |
| **Lymphocyte** | 29.7 | % | 16.5–49.5 | **Anti-SSA/Ro Ab** | <1.0 | U/mL | <10.0 |
| **RBC** | 3.73 | ×10^6^/μL | 3.86–4.92 | **Anti-SSB/Ro Ab** | <1.0 | U/mL | <10.0 |
| **Hb** | 10.2 | g/dL | 11.6–14.8 | **Aspergillus Ag** | Negative | NA | Negative |
| **PLT** | 15.7 | ×10^4^/μL | 15.8–34.8 | **Cryptococcus Ag** | Negative | NA | Negative |
| **CRP** | 4.37 | mg/dL | 0.00–0.14 | **T-SPOT.TB** | Negative | NA | Negative |
| **ESR** |  |  |  | **Anti-MAC antibody** | Negative | NA | Negative |
|  |  |  |  |  |  |  |  |
| **CD3^+^ T cells (% lymphocytes)** | 52.1 | % | 58–84 | **CD8^+^ T cells (% lymphocytes)** | 44.3 | % | 17–44 |
| **CD4^+^ T cells (% lymphocytes)** | 16.2 | % | 25–56 | **CD56^+^ NKT cells (% lymphocytes)** | 14.2 | % | 10–38 |

ANA: antinuclear antibody, AMA: antimitochondrial antibody, Ig: immunoglobulin, ACPA: anti-citrullinated peptide antibody, MAC: Mycobacterium avium complex, Ag: antigen, Alb: albumin, ALP: alkaline phosphatase, γ-GTP: γ-glutamyl transpeptidase, ALT: alanine transaminase, AST: aspartate transaminase, BUN: blood urea nitrogen, Cre: creatinine, CRP: C-reactive protein, Hb: hemoglobin, LDH: lactate dehydrogenase, PLT: Platelets, RBC: red blood cells, RF: rheumatoid factor, T-Bil: total bilirubin, TP: total protein, WBC: white blood cells.
