# Supplementary material for: Case Report: Novel IRF2BP2 variant in a Japanese patient with impaired B-cell differentiation, Th1 polarization, and systemic immune dysregulation
Source: Front Immunol. 2025 Oct 30;16:1662899. doi: 10.3389/fimmu.2025.1662899 (PMC12611837; doi:10.3389/fimmu.2025.1662899)
Supplement: Supplementary file 2 [file Table2.docx]

**Supplemental Table 2.** Clinical and immunological features of patients with IRF2BP2 deficiency.

|  | **Feature** | **Reported literature for IRF2BP2 deficiency*** | **Present case** |
| --- | --- | --- | --- |
| **Clinical manifestations** | | | |
|  | Recurrent sinopulmonary infections | Frequent | Yes |
|  | Bronchiectasis | Common | Yes |
|  | Recurrent fever | Reported | No |
| **Immunological features** | | | |
|  | Hypogammaglobulinemia | Frequent | Yes |
|  | Reduced memory B cells | Consistently reported | Yes |
|  | Impaired plasmablast differentiation | Consistently reported | Yes |
|  | Elevated interferon signature genes | Reported (Palmroth et al.) | Yes (IFI44L, LY6E, MX1) |
|  | STAT1 hyperactivation | Reported (Palmroth et al.) | Yes (inferred from IFN signature) |
|  | Th1 polarization | Not previously reported | **Yes (novel finding)** |
|  | Tfh expansion | Not previously reported | **Yes (novel finding)** |
|  | CD4+ lymphopenia | Reported | Yes |
| **Inflammatory manifestations** | | | |
|  | Inflammatory skin involvement | Reported | No |
|  | Inflammatory gastrointestinal involvement | Reported | No |
|  | Inflammatory bowel disease/colitis | Reported | No |
|  | Rheumatoid arthritis | Reported (Keller et al.) | No |
|  | Spondyloarthritis | Reported (Garcia-Aznar et al.) | No |
|  | Seronegative arthritis | Reported (Korholz et al.) | Yes |
|  | Thyroiditis | Reported (Udemgba et al.) | No |
|  | Pericarditis | Reported (Udemgba et al.) | No |
|  | **Primary biliary cholangitis** | **Not previously reported** | **Yes (novel finding)** |

*Summarized from Keller et al. J Allergy Clin Immunol 2016, 138(2):544-550; Garcia-Aznar et al. Front Immunol 2023, 14:1279171; Anim et al. Clin Immunol 2024, 266:110326; Udemgba et al. J Allergy Clin Immunol 2025, 155(6):2052-2062; Korholz et al. Rheumatology (Oxford) 2023, 62(4):1699-1705; Palmroth et al. Pharmaceuticals (Basel) 2021, 14(8).

**Bold text** indicates novel findings in the present case that expand the clinical and immunological spectrum of IRF2BP2 deficiency.
